# Candidate proteins from predegenerated nerve exert time-specific protection of retinal ganglion cells in glaucoma

**DOI:** 10.1038/s41598-017-14860-5

**Published:** 2017-11-06

**Authors:** Marita Pietrucha-Dutczak, Adrian Smedowski, Xiaonan Liu, Iwona Matuszek, Markku Varjosalo, Joanna Lewin-Kowalik

**Affiliations:** 1 Chair and Department of Physiology, School of Medicine in Katowice, Medical University of Silesia, Medykow 18, 40-752 Katowice, Poland; 2 Institute of Biotechnology, P.O. Box 65 University of Helsinki, 00014 Helsinki, Finland; 3 Department of Ophthalmology, School of Medicine in Katowice, Medical University of Silesia, Ceglana 35, 40-515 Katowice, Poland

## Abstract

Glaucoma is thought to be the main cause of severe visual impairment or permanent loss of vision. Current therapeutic strategies are not sufficient to protect against glaucoma. Thus, new therapies and potential novel therapeutic targets must be developed to achieve progress in the treatment of this insidious disease. This study was undertaken to verify whether the time of administration of an extract from predegenerated rat sciatic nerves as well as exposure time of this extract onto retinal ganglion cells (RGCs) influences the survival of RGCs in a rat glaucoma model. We have demonstrated that extract obtained from the predegenerated sciatic nerves protects RGCs in a rat glaucoma model. The neuroprotective effect depends mostly on the time of administration of the extract and less clearly on the time of exposure to the extract and is associated with stimulation of endogenous BDNF expression both in RGCs and glial cells. The 14^th^ day following glaucoma induction represents a therapeutic window for effective treatment in a glaucoma model. Mass Spectrometry analysis demonstrated that metallothionein 2 (MT2) may be a key molecule responsible for neuroprotective effects on RGC survival.

## Introduction

**Figure 1:**
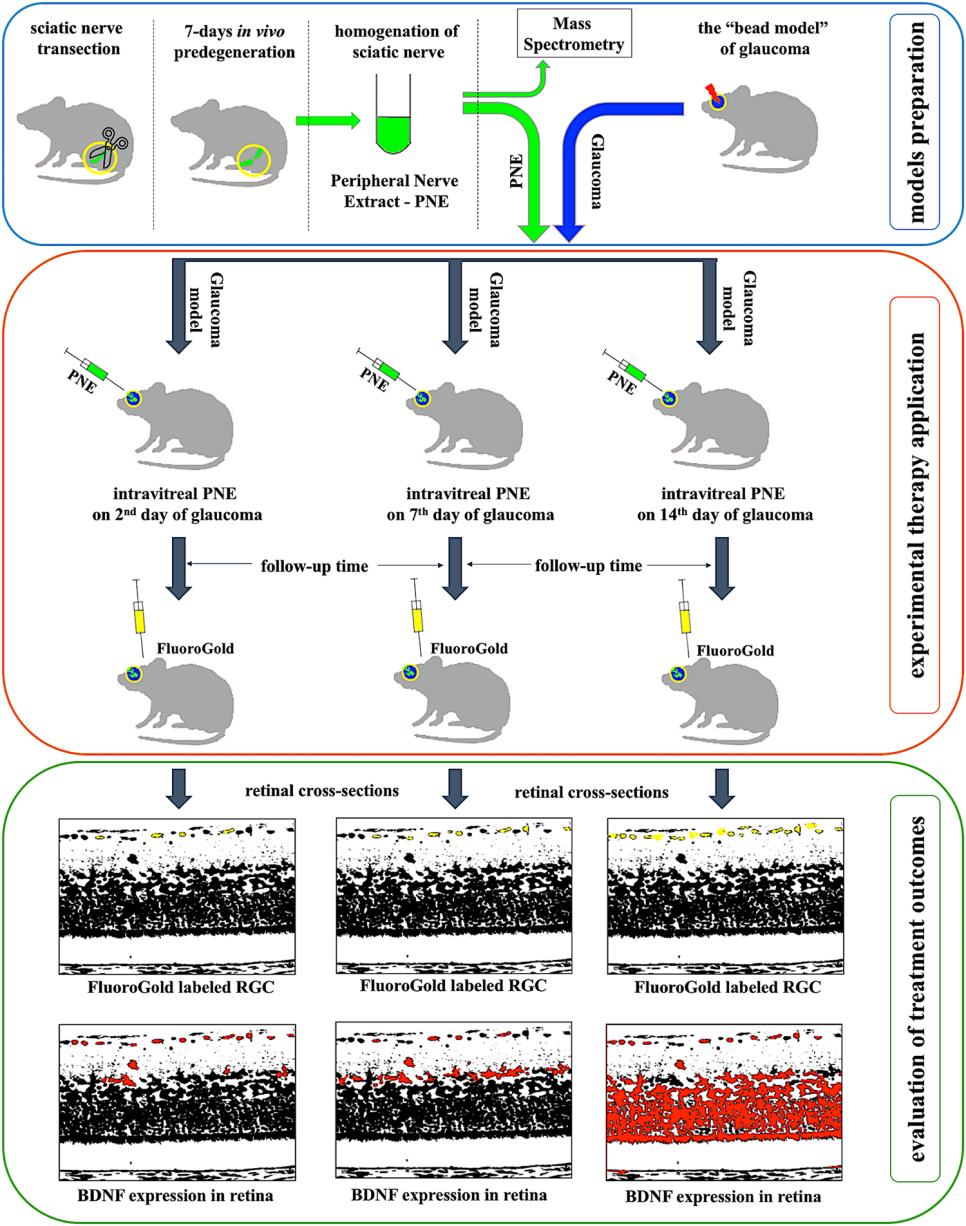
Graphical abstract.

Glaucoma is thought to be the main cause of severe visual impairment or permanent vision loss. This type of optic neuropathy is characterized by damage to RGCs and their axons^1^. RGC death is most commonly a consequence of elevated intraocular pressure (IOP), which has made lowering IOP, up to now, considered to be the most effective treatment method. However, it is known that high IOP is not the only pathological factor. One third of glaucoma patients with a normal IOP will develop typical signs and symptoms of glaucoma (i.e., NTG – normal tension glaucoma) and this ratio is even higher in the Asian population^2^. On the other hand, about eight times more prevalent than glaucoma is ocular hypertension (OHT) without optic nerve damage, suggesting that glaucoma might be a primary optic nerve disease that makes RGCs more prone to respond to risk factors such as increased IOP^3^. Thus, glaucoma is not a homogenous group of disorders; rather, it is believed that factors other than elevated IOP may be involved in the pathogenesis of this disease. Recent studies indicate that glaucomatous damage can also be related to inflammatory processes, oxidative stress, metabolic abnormalities and blood flow disturbances, as well as autoimmune responses^4–6^. An important issue is also the fact that the optic nerve is exposed to intracranial pressure, as it is surrounded by the cerebrospinal fluid (CSF) in the subarachnoid space. Thus, there is a new hypothesis that CSF circulatory dysfunction may play a role in the development of glaucoma^7,8^. It therefore seems that new therapies and potential novel therapeutic targets must be developed to achieve progress in the treatment of this insidious disease. Prospecting of new targets for IOP lowering, molecules modulating ocular haemodynamics and treatments providing neuroprotection for RGCs are future promising strategies^9^. Furthermore, because RGC and the optic nerve are parts of the central nervous system (CNS), glaucomatous neuropathy is considered to share similar features with other neurodegenerative diseases (i.e., Alzheimer’s disease)^10–14^ (Fig. 1).

Our previous studies revealed that purified extracts obtained from predegenerated rat sciatic nerves (PNE) stimulate outgrowth of damaged neurites in both the peripheral nervous system (PNS) and CNS^15–22^. The greatest neurotrophic activity was proved for fractions obtained from nerves predegenerated for 7 days^15–17^. For this reason, we decided to use similar extract to verify its therapeutic activity in a glaucoma model and test whether the time-point of administration of extract as well as exposure time of this extract onto RGCs influences survival of these cells. Furthermore we examined changes in the expression of endogenous BDNF after application of extract. We used our recently developed rat glaucoma model, which allows long-lasting IOP elevation with chronic damage of RGCs^23,24^. Moreover, we have analysed the protein composition of the extract from nerves predegenerated for 7 days by mass spectrometry and compare to the extracts obtained from non-predegenerated nerves as well as the nerves following longer-lasting predegeneration (i.e., 14 days). This study allowed us to identify candidate proteins that have possible protective effects on RGCs in the glaucoma model, from among which we chose MT2 as a potential neuroprotective agent identified in short-term predegenerated nerves. Additionally, we performed the screening via an *ex vivo* test using a retinal explant culture model.

## Results

### Short-term predegenerated peripheral nerve extract contains trophic factors and antioxidants – proteomic data

Mass Spectrometry analysis of sciatic nerve extracts revealed differences in the proteomic map between short- and long-term predegeneration. Short-term predegeneration (up to 7 days *in vivo*) was associated with presence of proteins potentially important for neuroprotective and neuroregenerative effects (Supplementary Table 1). This justifies usage of peripheral nerve extract of short-term predegeneration in therapies focusing on neuroprotection. Unique candidate proteins detected in short-term predegenerated samples are listed in Table 1.

**Table 1:** Candidate proteins identified in mass spectrometry analysis in sciatic nerve homogenate.

Candidate protein	Control nerve	Short-term predegeneration	Long-term predegeneration	Comments	Referrence
ELAVL1/HuR	0	+	0	RNA binding protein	Brennan *et al*.^62^
					Bolognani *et al*.^63^
					Skliris *et al*.^64^
metallothionein 2 (MT 2)	0	+	0	antioxidant	Santos *et al*.^78^
				neuroprotection	
				neuroregeneration	
cilliary neurotrophic factor (CNTF)	+	+	0	neuroprotection	Wen *et al*.^57^
				neuroregeneration	
glial maturation factor γ (GMF)	0	+	0	neurotrophic factor	Lim and Huang^59^
				neural development	
				neuroregeneration	
dynamin-2	0	+	0	axonal growth	González-Jamett *et al*.^65^
nucleolin	0	+	0	oxidative stress defence	Caudle *et al*.^66^

These proteins represent a family of RNA-binding proteins, intracellular antioxidants and growth factors involved in cell survival and regeneration. Since MT2 seems to represent the strongest neuroprotective potential towards RGCs in glaucomatous conditions, we decided to use this protein in further screening tests using retinal explant cultures to verify its properties.

**Figure 2:**
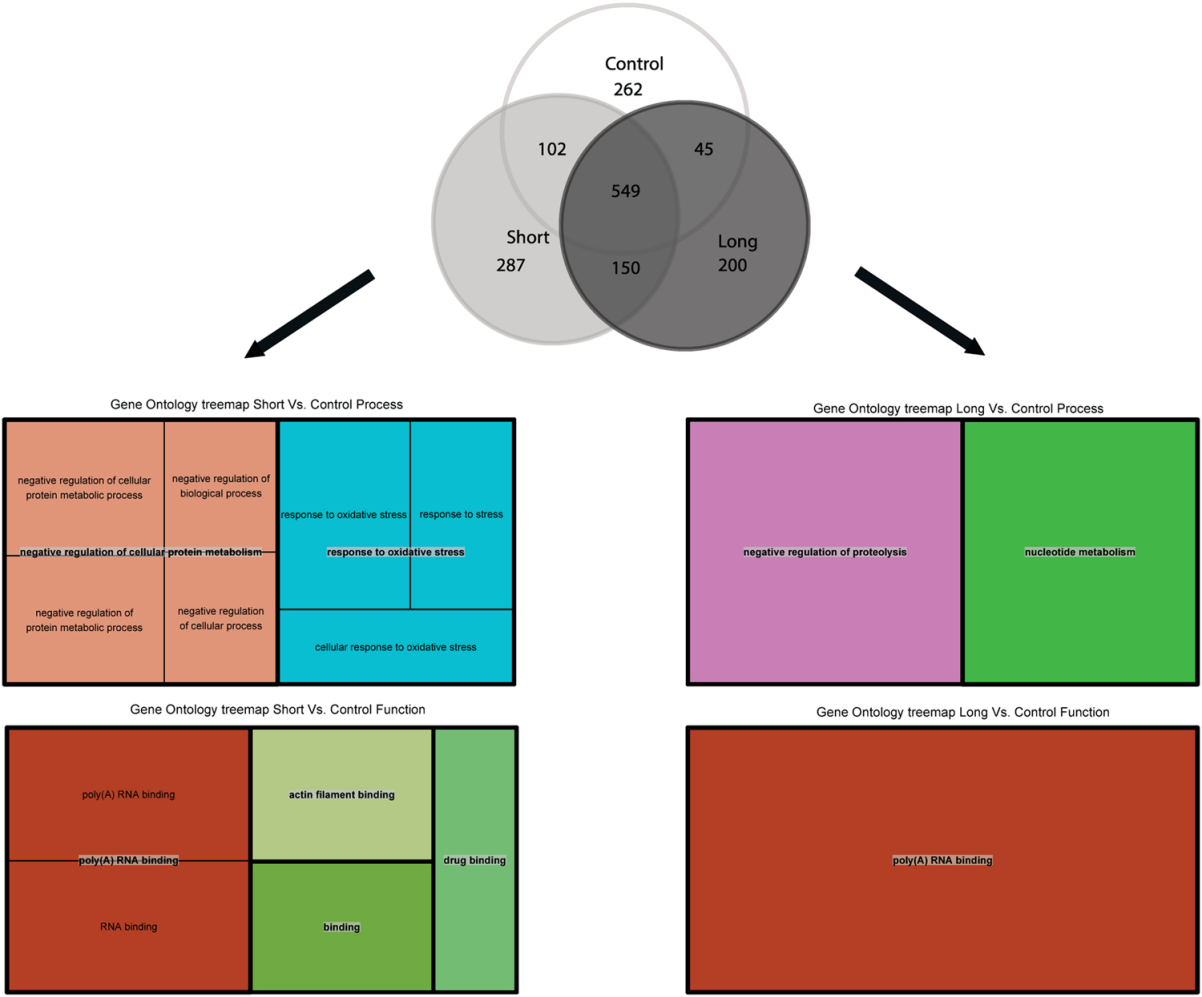
GOrilla gene ontology enrichment analysis, TreeMaps from REVIGO for GOslim biological process and molecular function. Differences between short- and long-time predegenerated nerve extracts in comparison to control one are presented as clusters. Each rectangle in TreeMap represents a single cluster. The joined ‘superclusters’ include loosely related terms. The size of the rectangles reflects the p value.

Gene ontology enrichment analysis showed differences between short- and long-term predegenerated nerve extracts in comparison to controls. Enriched clusters in short-term predegenerated nerves were represented mostly by oxidative stress response proteins, negative regulators of protein metabolism and RNA binding proteins, while in long-term predegenerated nerves the difference was much less pronounced (Fig. 2; Supplementary Table 2).

### The number of FluoroGold-positive (FG-positive) retinal ganglion cells

**Figure 3:**
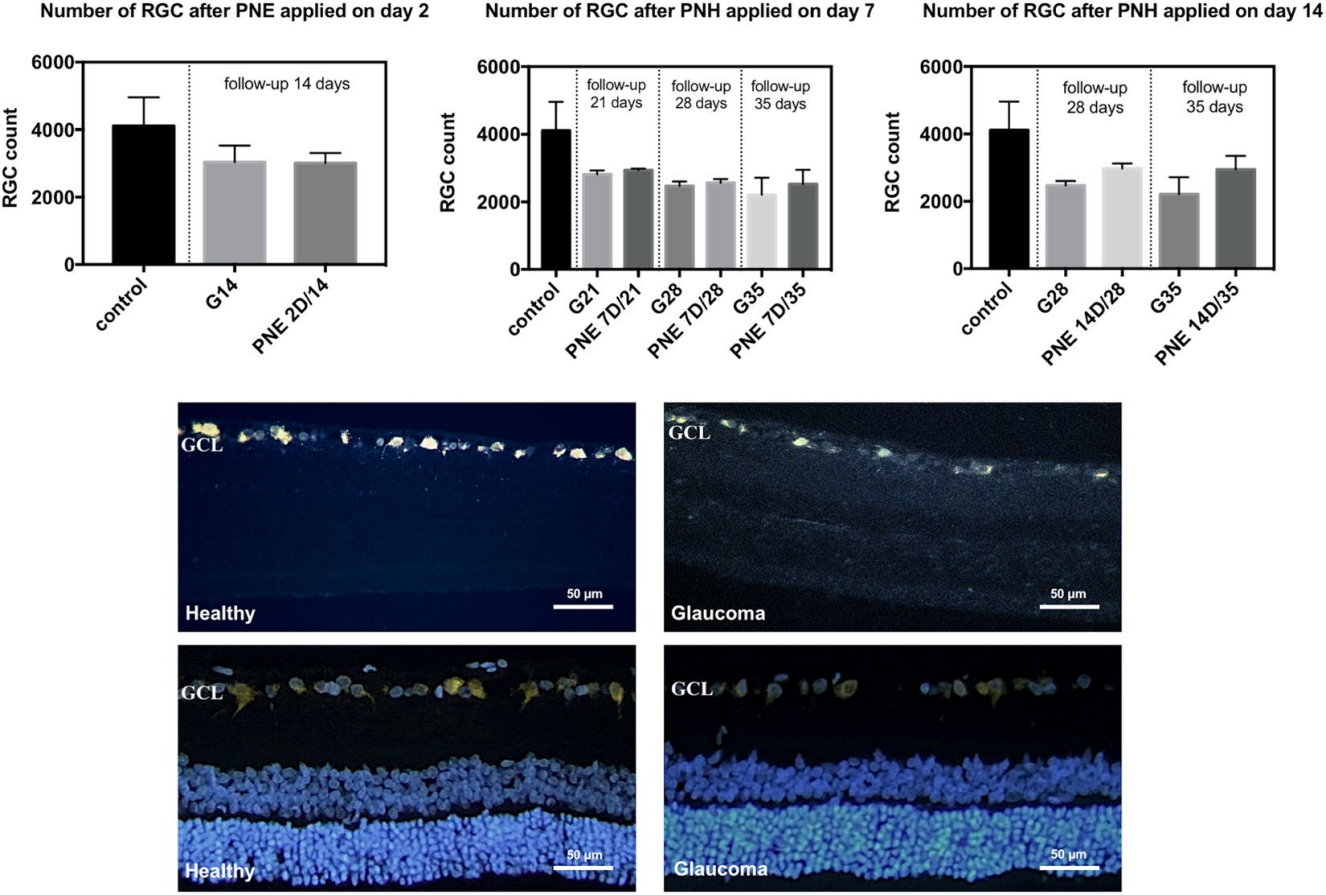
Retinal ganglion cells count (for FluoroGold-positive cells) in experimental groups. Control – healthy; G14/G21/G28/G35 – glaucoma with PBS injection, euthanasia on 14^th^, 21^st^, 28^th^ or 35^th^ day respectively; PNE 2D/14 – glaucoma with PNE injection on the 2^nd^, euthanasia on the 14^th^ day; PNE 7D/21 - glaucoma with PNE injection on the 7^th^, euthanasia on the 21^st^ day; PNE 7D/28 - glaucoma with PNE injection on the 7^th^, euthanasia on the 28^th^ day; PNE 7D/35 - glaucoma with PNE injection on the 7^th^, euthanasia on the 35^th^ day; PNE 14D/28 - glaucoma with PNE injection on the 14^th^, euthanasia on the 28^th^ day; PNE 14D/35 - glaucoma with PNE injection on the 14^th^, euthanasia on the 35^th^ day. PNE – Peripheral Nerve Extract. Retinal cross-sections: in upper panel represent FluoroGold labeled RGC in GCL of healthy and glaucomatous retinas; in lower panel represent FluoroGold and DAPI labeled RGC in GCL of healthy and glaucomatous retinas. GCL–Ganglion Cells Layer; RGC–Retinal Ganglion Cells.

In all experimental groups in which PNE was applied, we observed that the number of FG-positive RGCs was higher than in phosphate buffered saline (PBS)-treated groups. This relation was the stronger the longer observation time was applied. Only in the group PNE 2D/14 (3008 ± 300 cells) were the results comparable to those obtained in the PBS group (G/14 – 3034 ± 489 cells) (p > 0.05) (Fig. 3).

### The relationship between the survival of RGCs and time-point of peripheral nerve extract administration

**Figure 4:**
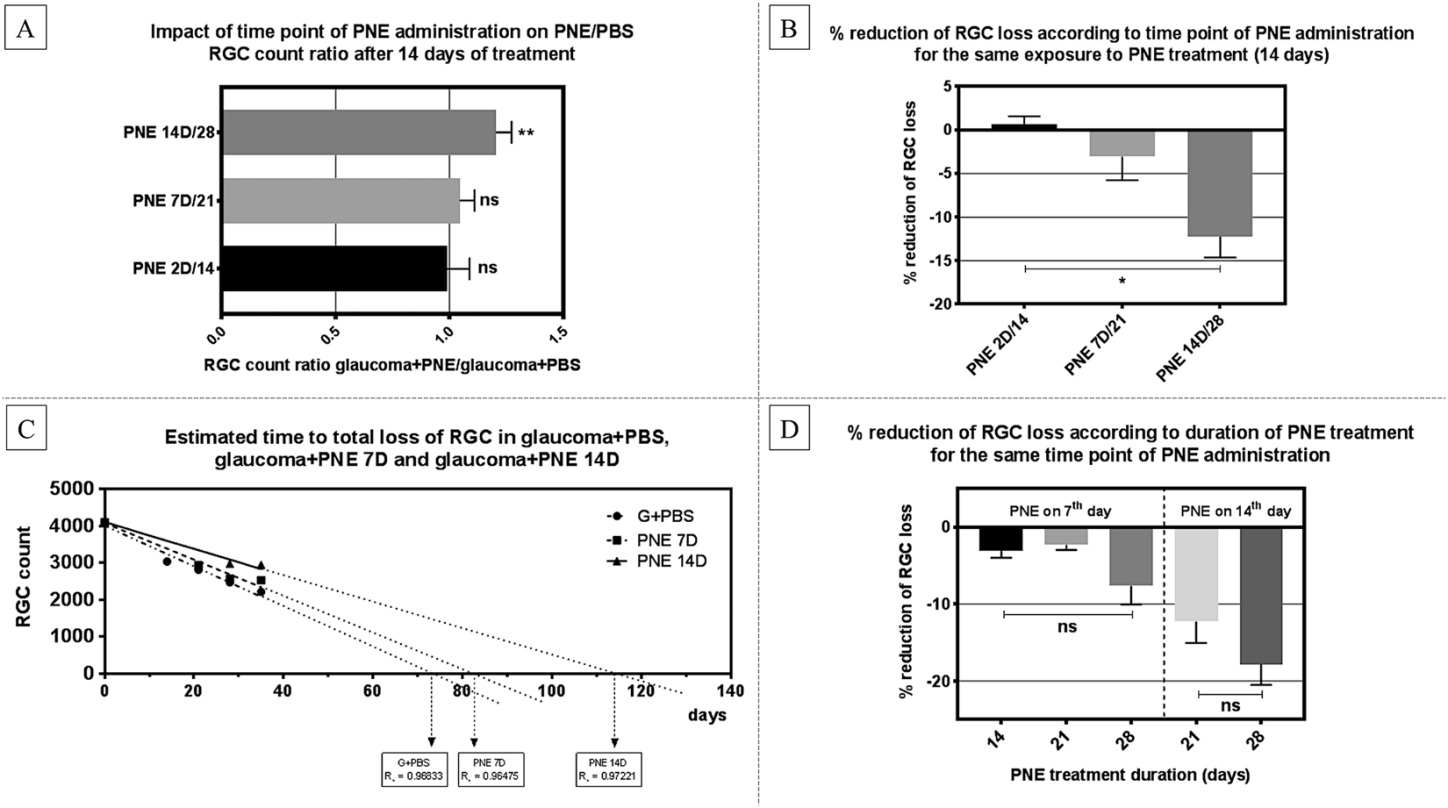
(**A**) Treated/non-treated RGC count ratio in groups exposed to PNE for 14 days with different PNE injection time-point. (**B**) Reduction of RGC loss (%) in groups exposed to PNE for 14 days with different PNE injection time-point. (**C**) Linear regression model with extrapolation of RGC survival in groups injected with PNE on day 7 and day 14. (**D**) Reduction of RGC loss (%) in groups injected on the same day (day 7 or 14), exposed to PNE for 14 days or longer. Statistical analysis indicated with: ns – non-significant, single asterisk (*) – p < 0.05, double asterisk (**) – p < 0.03. PNE – Peripheral Nerve Extract; RGC – Retinal Ganglion Cells.

To determine if the time-point of PNE injection influenced RGC survival, we compared RGC survival ratio between groups with similar exposure time to PNE (i.e., 14 days) that received PNE on the 2^nd^, 7^th^ or 14^th^ day from glaucoma induction with comparison to PBS-injected controls. The greatest reduction of RGC loss was observed in group that received PNE on the 14^th^ day following glaucoma induction (PNE 14D/28 – RGC loss 27.6% vs G28 – RGC loss 39.8%; survival of RGC better for 12.2%), then in the groups where extract was administered on the 7^th^ (PNE 7D/21 – RGC loss 28.5% vs G21 – RGC loss 31.5%, survival of RGC better for 3%) and 2^nd^ days (PNE 2D/14 – RGC loss 26% vs G14 – RGC loss 26%, no benefit in RGC survival). To estimate the long-term survival of RGCs, we built a linear regression model of RGC survival. In this simulation, constant RGC loss in glaucoma was presumed to model the general tendency of the glaucomatous course. We observed that in our model, after PNE was administered on the 7^th^ day, the total loss of RGCs would appear after approximately 85 days (compared to 75 days with PBS treatment). Injection of PNE on the 14^th^ day would prolong this time up to nearly 120 days (Fig. 4A–C).

### The relationship between the survival of RGCs and exposure time to peripheral nerve extract

To determine if the exposure time to the PNE affects RGC survival, we compared RGC survival ratio between groups with the same time-point of PNE injection (i.e., 7^th^ day or 14^th^ day) and with different follow-up times (14 days or 21 days) with comparison to PBS-injected controls. In group that received PNE on the 14^th^ day after glaucoma induction, the reduction in RGC loss after 14 days of exposure to PNE was at the level of 12.2% when compared to the corresponding PBS-treated group, and after 21 days was at the level of 17.7%. In the group that received PNE on 7^th^ day after glaucoma induction, the reduction in RGC loss after 14 days of exposure to PNE was at the level of 3.1% when compared to the corresponding PBS-treated group; after 21 days it was at the level of 2.3% and after 28 days at the level of 7.6%. We must consider that RGCs in PNE 7D were exposed to elevated pressure for 7 days, while in PNE 14D for 14 days before PNE supplementation had been used (Fig. 4D).

### Endogenous brain derived neurotrophic factor (BDNF) distribution showed alterations depending on time-point of peripheral nerve extract injection

**Figure 5:**
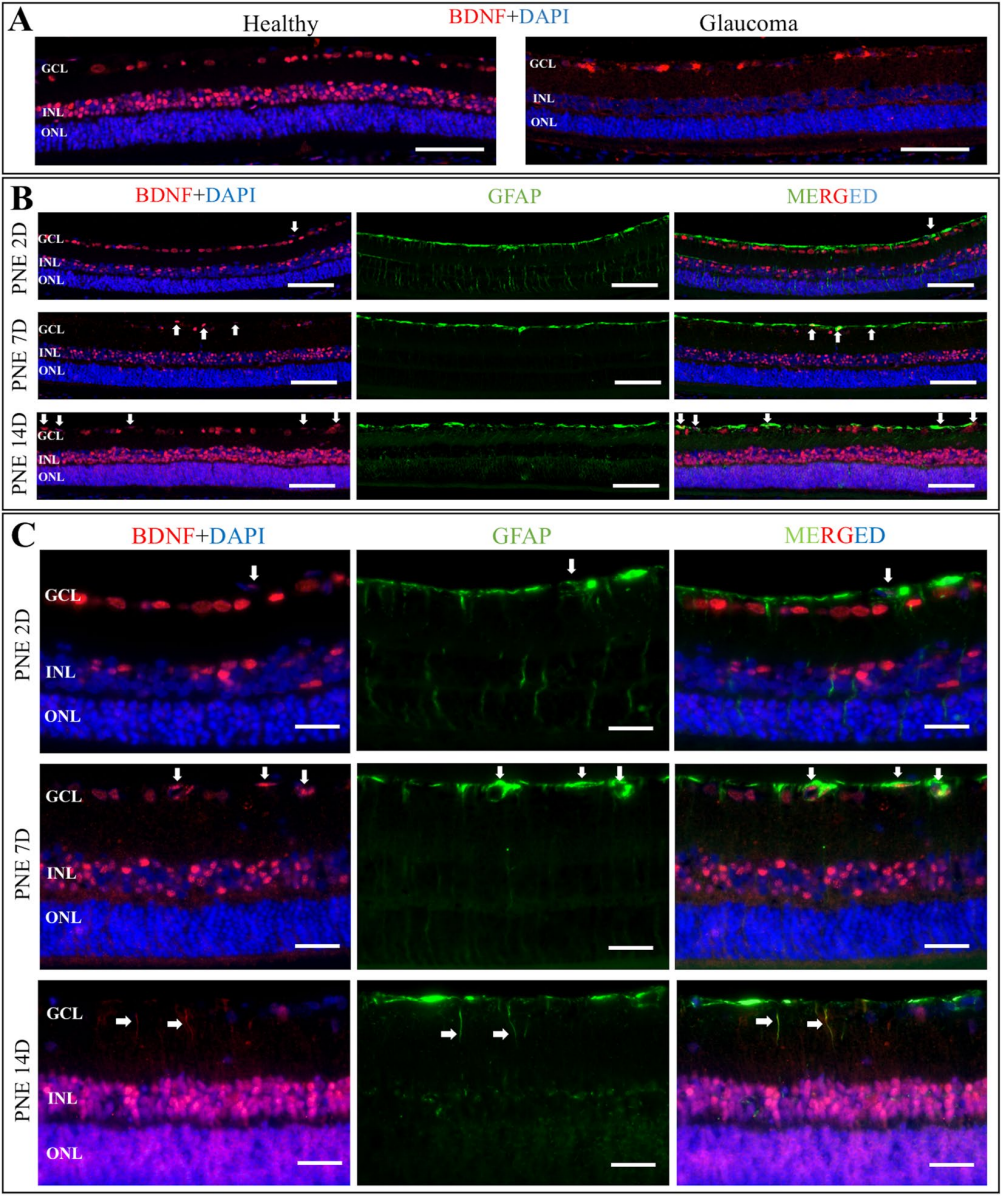
Representative retinal cross-sections immunostaining with anti-BDNF (red) and anti-GFAP (green); DAPI (blue). (**A**) Panel showing localization of BDNF expression within healthy and glaucomatous retina. Glaucomatous damage is associated with generalized decrease in BDNF expression in all retinal layers. Scale bar = 200 μm. (**B**) Panel showing co-localization of BDNF and GFAP in glaucomatous retina after certain PNE treatment protocols. PNE 2D – the extract injected on the second day after glaucoma induction, incubation time 14 days. PNE 7D - the extract injected on the seventh day after glaucoma induction, incubation time 14 days. PNE 14D - the extract injected on the fourteenth day after glaucoma induction, incubation time 14 days. Different time-point of PNE injection resulted in different expression of BDNF in retinal layers. The strongest expression of BDNF was observed when PNE was injected on 14^th^ day after glaucoma induction. Arrows point out merged co-localization of BDNF with GFAP marker indicating BDNF expression in retinal glia nuclei. Scale bar = 200 μm. (**C**) Panel showing localization of BDNF expression in different compartments of retinal glial cells that depend on time-point of PNE injection. The later administration of PNE the more increased expression of BDNF was observed. Additionally, to nuclear glial expression of BDNF noticeable after PNE injection (white arrows), administration of PNE on day 14 resulted specifically in BDNF staining within glial cells processes (white arrows). Scale bar = 50 μm. PNE – Peripheral Nerve Extract; BDNF – Brain Derived Neurotrophic Factor; GFAP–Glial Fibrillary Acidic Protein; GCL – Ganglion Cells Layer; INL – Inner Nuclear Layer; ONL – Outer Nuclear Layer.

In all glaucomatous retinal cross-sections, the difference in activation and proliferation of glial cells, as the typical sign of glaucomatous damage, was observed. These changes were noticeable independently from extract supplementation, but their intensity depended on the exposure time to high IOP (the duration time of the experiment) and on day in which the extract was injected (Fig. 5).

In our experiment, BDNF was highly expressed in inner nuclear layer (INL) and ganglion cell layer (GCL) in the healthy eyes compared with the sections from glaucoma eyes, where weaker expression of this neurotrophin was observed (mainly in GCL layer). BDNF expression in the group where the extract was injected on the 2^nd^ day after glaucoma induction was lower than in healthy eyes, but slightly higher than in the glaucoma control eyes. Changes in BDNF expression could be more clearly observed when the extract was served later, particularly on the 14^th^ day. In this group, BDNF expression was exceptionally higher in both INL and outer nuclear layer (ONL) than in GCL. Furthermore, BDNF expression in GCL, INL and ONL was co-localized with glial cell markers – glial fibrillary acidic protein (GFAP) and was present in both – cell bodies and processes of glial cells, which were not perceived in the group with extract injection on the 7^th^ day, where BDNF was detected only in some of the bodies of glial cells (Fig. 5, Table 2).

**Table 2:** Qualitative analysis of BDNF expression in retinal cross-sections according to experimental groups (++ strong expression; +weak expression; +/− expression appears only in some cross-sections; − without expression).

	GCL	INL	ONL	GLIAL CELLS BODY	GLIAL CELLS PROCESSES
Healthy	++	+	+/−	+/−	−
Glaucoma	+	+/−	−	−	−
PNE 2D	++	+	+/−	+/−	−
PNE 7D	+/−	+	+/−	+	−
PNE 14D	+	++	++	+	+

### *Ex vivo* retinal explants culture

**Figure 6:**
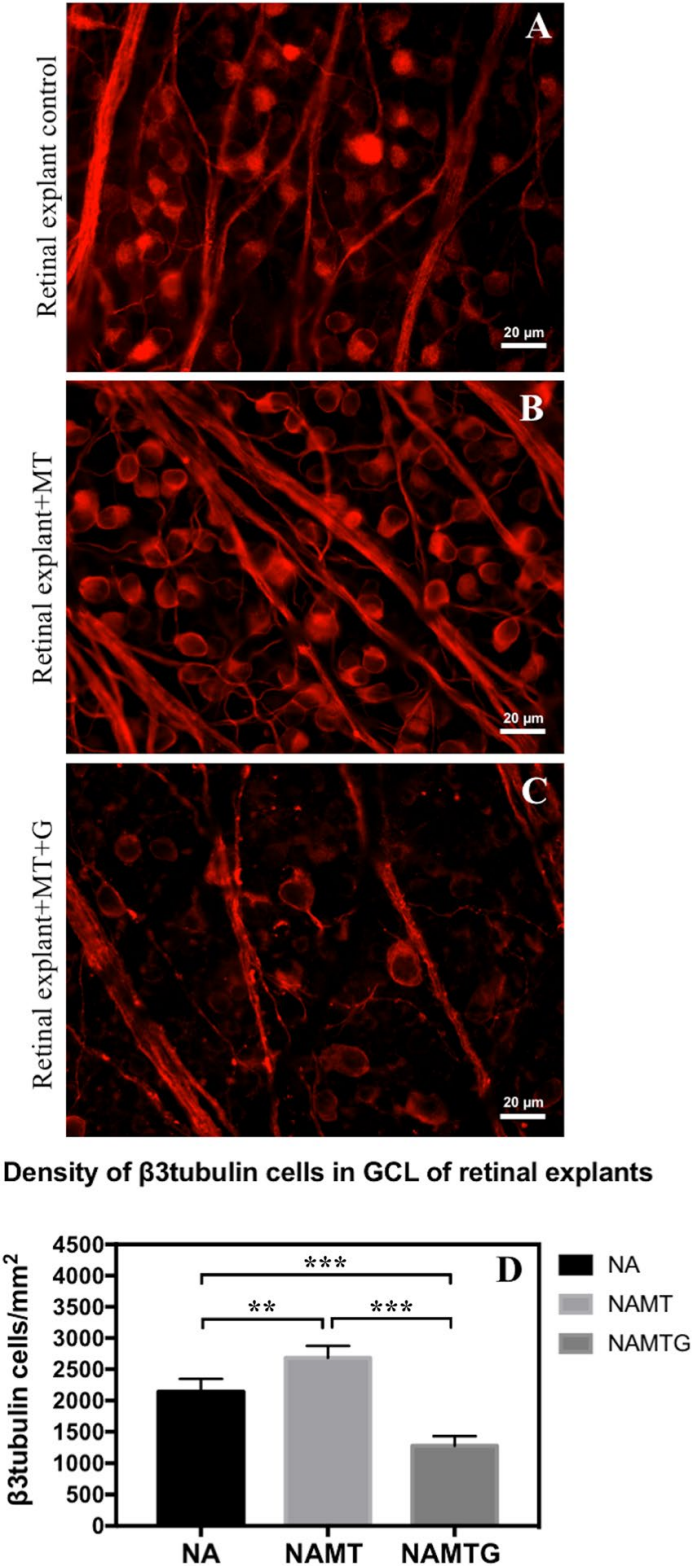
*Ex vivo* retinal explants cultured for 7 days in standard Neurobasal A medium with no treatment (**A**), treated with 1 µg/ml of Metallothionein II (**B**) and treated with 1 µg/ml of MT II and 10 µl/10 ml Gentamycin (**C**). Immunofluorescent staining for β3tubulin with AlexaFluor 594. Scale bar = 20 μm. NA – explants in standard Neurobasal A medium with no treatment; NAMT – explants treated with 1 µg/ml of Metallothionein II; NAMTG – explants treated with 1 µg/ml of MT II and 10 µl/10 ml Gentamycin; MT – metallothionein 2; G – Gentamycin. Statistical analysis indicated with: double asterisk (**) – p < 0.03, triple asterisk (***)–p < 0.005, independent t-test.

To perform the screening experiment on whether MT2 detected in a mass spectrometry analysis of short-predegenerated PNE has neuroprotective potential, we exposed *ex vivo* cultured rat retinal explants to this protein for 7 days. Density of β3tubulin positive cells in GCL was significantly higher in explants cultured in standard medium supplemented with MT2 (2688 ± 192 cells/mm^2^) than in explants in standard medium (2142 ± 203 cells/mm^2^). The lowest density of β3tubulin positive cells (1279 ± 152 cells/mm^2^) was reported in explants cultured in standard medium supplemented with MT2 and Gentamycin, which serves as a blocker of Megalin receptor that is linked to MT2 activity (Fig. 6).

## Discussion

In our previous studies, we proved that protein extract obtained from predegenerated rat sciatic nerves stimulates RGC survival and promotes neurite outgrowth in a model of acute optic neuropathy – axotomy^18,19^. It seemed reasonable that when we successfully developed the rat model of chronic optic neuropathy (glaucoma), we should undertake efforts to verify the further neurotrophic potential of this extract. Moreover, glaucoma, as an important cause of irreversible blindness, is much more common than axotomy of the optic nerve, so our research would have potential clinical significance. In the present study, we have shown that protein extract obtained from predegenerated nerves exerted a protective effect on RGC survival in a rat model of glaucoma. In each experimental glaucoma group that received the extract supplementation we noticed it’s protective impact on the RGC survival. It is important to note that the day on which extract was injected into the eye was essential. Reduction of RGC loss was significantly greatest when extract was applied on the 14^th^ day after glaucoma induction. This relation was observed independently from how long RGCs were exposed to the high IOP. Therefore, our results suggest that the time-point in which this treatment should be applied is pivotal.

RGC death is considered to be a biphasic process, in which the initial primary injury caused by the presence of excessive amounts of glutamate is responsible for the initiation of damage. Afterwards, secondary degeneration occurs and intracellular glutamate is released from the dying cells and dispersed among neighbouring cells, triggering a cascade of excitotoxicity events leading to further cell death^25,26^. Some reports distinguish up to five stages pointing glial cells as initiators of reactions leading to RGC death after IOP elevation^27^. Other studies notify that RGCs may be relatively invulnerable to glutamate and N-Methyl-D-aspartic acid (NMDA) excitotoxicity, which suggests that the cells around them—amacrine cells and Müller glia—are much more sensitive and that perhaps the primary damage to these cells stimulates RGC death as a secondary degeneration^28^. Thus, before RGC is subjected to irreversible apoptotic changes, we have time for effective neuroprotection. Our results show that such neuroprotection, when applied too early (i.e., 7 days following glaucoma) is not as effective as later administration (i.e., 14^th^ day). This time-point is a therapeutic window allowing for effective treatment in our rat glaucoma model. Reports of other researchers on both animals and humans also imply the existence of a time window between RGC dysfunction and RGC morphological loss^26,29–31^. Possibly the reason why early protection on the 7^th^ day is not as effective as on the 14^th^ day is because the primary changes in retinal environment, such as the expression of cytokines^32–34^ and the activation of neuroinflammatory and stress responses^35–37^ disturbs homeostatic balance, creating unfavourable conditions for the application of the treatment. Our observations suggest that the ocular environment, at least in our model, needs more than a week for the preparation of suitable conditions for such therapy. The key to success seems to be the question, what changes take place in the early stage following glaucoma induction?

*Johnson E*. observes as early as 3 days after IOP elevation the loss of the gap junctional connexin 43 labelling and astrocytic proliferation in the ONH, then (on the 7^th^ day) a decrease labelling of neurotrophins NT4/5 and BDNF both in the ONH and the inner layers of the retina^38^. Subsequently, depositions of collagen IV, collagen VI, and laminin are formed (11^th^ day)^39^. Therefore, these early changes impair the communication between distal axons and cell bodies of RGCs. The results of our study suggest that the cell communication disturbance in the early stages of glaucoma do not disqualify later therapeutic effect of extracts, perhaps indicating the reversibility of these changes. Interestingly, the same group of researchers reports that after 14 days of exposure to high IOP, neurotrophin labelling NT4/5 and BDNF returns to the nerve head glia and appears in RGCs^38^. This increased synthesis of BDNF may represent an endogenous neuroprotective response of RGCs, which was highlighted by *Vecino* ^40,41^. Lots of studies have reported that BDNF protects RGCs in various models of optic nerve injures and diseases^42–44^ and inhibits the osmotic swelling of Müller cells and bipolar cells^45^. Likewise, BDNF up-regulates the electrogenic glutamate-aspartate transporter (GLAST) and glutamine synthase (GS) expression in mouse retinal Müller cells increasing glutamate uptake during hypoxia^46^. Perhaps by applying extract on the 14^th^ day following glaucoma, we have supported or enhanced the endogenous neuroprotective system of cells. What is interesting, in our study, even without PNE treatment we observed the stronger expression of BDNF compared to healthy tissue but only in GCL. Application of PNE not only intensifies expression but also changes its location. After injection of the extract on the 14^th^ day we noted a strong expression of BDNF in both INL and ONL and a weaker expression in GCL, even if animals were sacrificed on 35^th^ day following glaucoma. Notably, BDNF was located not only in retinal neurons but also in both cell bodies and processes of glial cells.

It is commonly known that glia activation (including microglia) can be beneficial or detrimental to the local neurons. Depending on the type of damage or the duration of the response, microglia may secrete both anti- and pro-inflammatory cytokines^47–49^. There is the possibility that glia may disclose this detrimental nature on the 7^th^ day. Then anti-inflammatory cytokines downregulate the expression of pro-inflammatory cytokines, reactive oxygen species and chemokines, which could explain why injection of PNE later is more effective^50,51^. Furthermore, TNFα and IL-1 stimulate the synthesis of interleukin-6 (IL-6), which increases BDNF levels and enhancing the endogenous neuroprotective system of cells, about which we mentioned earlier^52,53^.

Thus, both our previous and current research unquestionably demonstrate the protective nature of PNE, but the key question remains: What components provide such properties? Mass spectrometry analysis comparing the composition of extracts from non-predegenerated sciatic nerves (control) and extracts after short (up to 7 days) and long predegeneration (14 days), enables us to indicate some promising candidate proteins. The neuroprotective effect of ciliary neurotrophic factor (CNTF) on RGCs and photoreceptors is commonly known^54–57^; thus, the presence of this protein in the extract is not surprising. Undoubtedly, this protein is conferring certain protective features of the extract, but is not the major contributor deciding about especially advantageous properties of the extract after short-term predegeneration. It therefore seems that the presence of this protein in predegenerated extracts may render them therapeutic properties, however does not explain the beneficial effects of short-term predegenerated extract. Therefore, we decided to attend to these proteins, which are present only in extracts after short-term predegeneration and are absent after long predegeneration and in extracts without predegeneration. Human antigen R (HuR), MT2, glial maturation factor (GMF), dynamin-2 and nucleolin are proteins that are present only in the extract after short-term predegeneration. Considering our previous studies in which we found that the extracts obtained from nerves predegenerated for 7 days have the extremely high neuroregenerative and neuroprotective potential, the appearance of these proteins in such extracts may suggest their participation in these processes. GMF is expressed by Schwann cells of distal segments of the transected nerve 3 days after axotomy, gaining the peak expression on the 7^th^ day^58^. It is believed that GMF, as a growth and differentiation factor, is acting on neurons as well as glia, and can participate in the regeneration in the CNS^59^. On the other hand, recent studies suggest that this factor is associated with the pathophysiology of neurodegenerative disorders, such as Alzheimer’s disease^60,61^. Embryonic lethal abnormal vision-like 1/Human antigen R (ELAVL1/HuR) protein binds to A-U rich areas of mRNA and regulates mRNA stability. Because of this protective activity, ELAV family proteins accompany targeted mRNA on the pathways from nucleus to cytoplasm and act, in the majority of cases, as a positive regulator of gene expression^62,63^. HuR protein is involved in the regulation of expression of proteins participating in cellular stress response (i.e., heat shock protein - Hsp70). Recent studies showed that HuR^−/−^ knockout animals develop hypersensitivity of hippocampal neurons to oxidative and excitotoxic damage^64^. Dynamin-2 and nucleolin are proteins involved in axonal outgrowth and neuronal oxidative stress defence. Dynamin-2 dysfunction can participate in Alzheimer’s disease development by impairing endocytosis of ß amyloid, resulting in its cellular accumulation^65^. Nucleolin, a novel protein discovered in Parkinson’s disease development, has a selective specificity for oxidative stress and proteasomal pathways. Experimental manipulation of nucleolin levels in a cellular model of Parkinson’s disease induces an altered sensitivity to the selected neurotoxic effects^66^. An especially interesting protein seems to be MT2, which as a secondary antioxidant cooperates with reduced glutathione (GSH) in the cellular protective system against oxidative stress^67,68^. Glutathione disulfide (GSSG) oxidizes MT, while GSH reduces the oxidized protein to thionein, which then binds to available zinc^68^. Exogenous administration of MT2 leads to an increase in regenerative sprouting in dorsal root ganglion after axotomy^67^ and inhibits the p38 and proinflammatory cytokines expression such as interleukin-1β (IL-1β), IL-6 and TNFα in post-burn inflammation^69^. Levels of MT2 decrease with ageing, implying a role of these proteins in the development of age-related neurodegenerative pathologies, i.e., Alzheimer’s and Parkinson’s diseases^70^ and retinal diseases such as age-related macular degeneration (AMD) and retinitis pigmentosa (RP)^71^. It is observed that MT2 may contribute to the prolonged survival of photoreceptors in the course of retinal degeneration and stimulates neurite growth of RGC involving megalin, which is present both in the inner and the outer layer of the retina^72–74^. Additionally, MT2 can induce increases in endogenous BDNF expression, which we also observed in our study. The above data and the presence of MT2 in 7-day predegenerated extract could suggest that this protein may be responsible for the beneficial properties of the extract. To verify whether MT2 could be applied in glaucoma treatment, we decided to perform the screening test using retinal explant culture. Our results clearly indicate that MT2 shows neuroprotective effects towards RGC.

In conclusion, we have demonstrated that extract obtained from the predegenerated sciatic nerves protects RGCs in a rat glaucoma model. Furthermore, the success of neuroprotective treatments depends mostly on the administration time of the extract and less clearly on the exposure time to the extract. In our study, 14 days following glaucoma induction represents a therapeutic window allowing for the initiation of effective treatment in a glaucoma model. Additionally, our findings note that MT2 may be a key molecule responsible for such beneficial effects of PNE on RGC survival. However, it is also possible that MT2 reveals its therapeutic profile in combination with other substances present in the extract.

## Material and Methods

### Animals and anaesthetics

The experiments were performed on 8-week old male Wistar rats, weighing approximately 200 g. All animals were provided by the Center for Experimental Medicine, Medical University of Silesia, Katowice, Poland and were treated in accordance with the ARVO Statement for the Use of Animals in Ophthalmic and Vision Research and the EC Directive 86/609/EEC for animal experiments, using the protocols approved and monitored by the Local Committee for Animal Experiments of Medical University of Silesia.

All surgical procedures were performed under general anaesthesia with an intraperitoneal injection of a mixture of ketamine (50 mg/kg, VetaKetam, Vetagro, Poland) and xylazine (5 mg/kg, Xylapan, Vetoquinol Biowet, Poland). For the local anaesthesia, we used 0.5% Proxymetacaine hydrochloridum (Alcaine, Alcon, Fort Worth, TX, US). During the recovery from anaesthesia, the rats were placed in their cages and an ointment containing ofloxacin (Floxal, Bausch&Lomb, Bridgewater, NJ, US) was applied on the cornea to prevent corneal desiccation and infection. After follow-up time, the animals were sacrificed with an intraperitoneal overdose of a mixture of ketamine and xylazine.

### Glaucoma model

**Figure 7:**
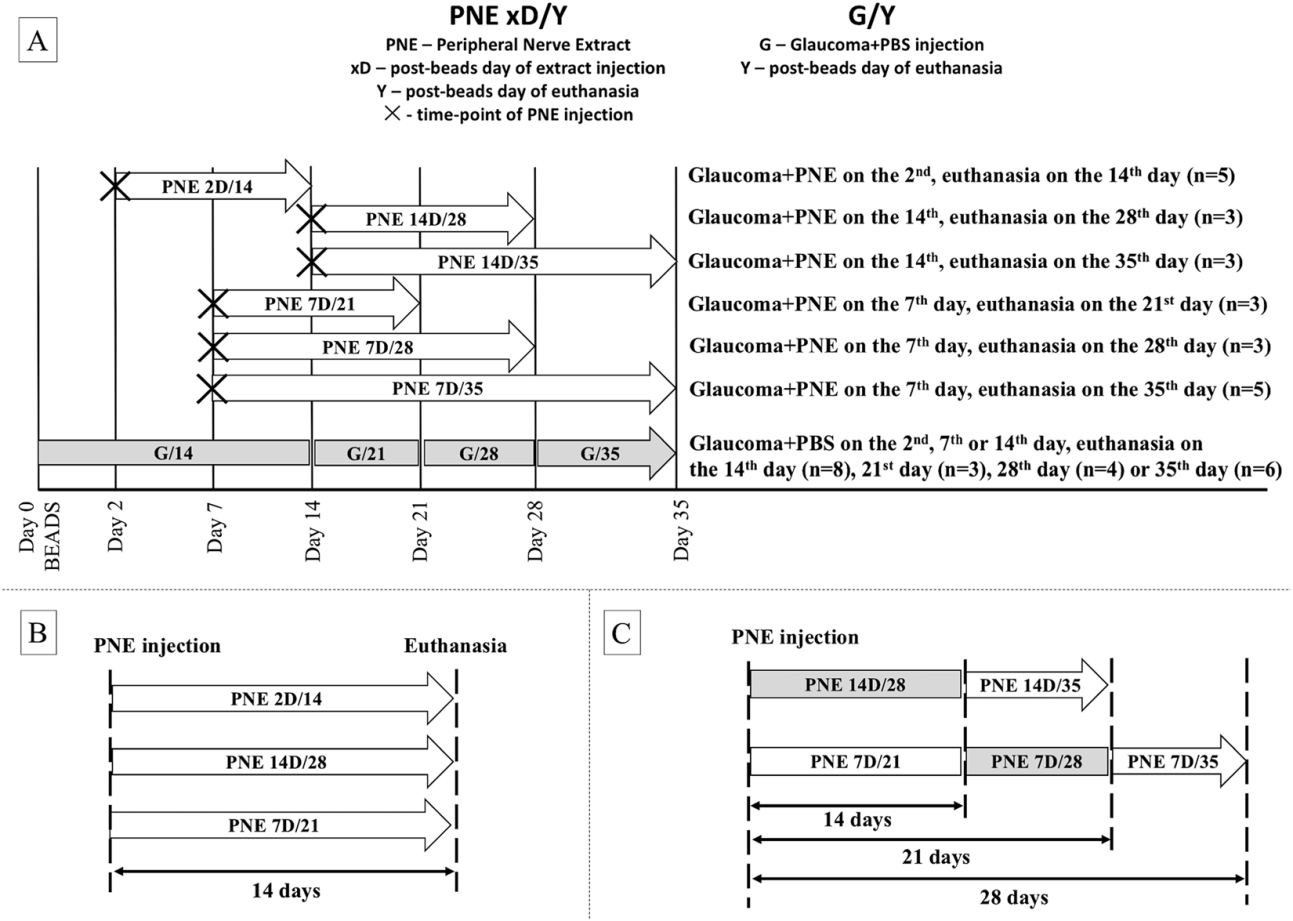
(**A**) Experimental groups, arrows indicate duration of PNE treatment. Basic treatment time was 14 days, PNE was injected in different time points (2, 7, 14 days) of ocular hypertension to evaluate impact of time of injection on RGC survival. Then PNE was injected on same time point (7 or 14 day) and duration of PNE treatment was extended (up to 21 or 28 days post-injection) to analyze PNE effect in time. (**B**) The same PNE treatment duration (14 days), but PNE injected on different time points of glaucomatous damage (2, 7 or 14 days). Loss of RGC compared to PBS-treated glaucoma from the same time points of damage. (**C**) PNE injected on the same time point of glaucomatous damage (7. day or 14. day), but with different duration of treatment (14 days or >14 days). Loss of RGC compared to PBS-treated glaucoma from the same time point of damage. PNE – Peripheral Nerve Extract; PBS – phosphate buffered saline; RGC – Retinal Ganglion Cells.

Experimental glaucoma was induced unilaterally (the left eye) based on modified bead model with initial high pressure injury, as described before^23,24^. We used forty-three male Wistar rats (see detailed description of subgroups on Fig. 7). Briefly, animals were anesthetized generally and topically, and a glass microcapilar was used for intracameral injection of 15 μl suspension consisting of 5 μl polystyrene microbeads of 10.0 μm diameter followed by 5 μl microbeads of 6.0 μm diameter (Polybead Microspheres; Polysciences, Inc., Warrington, PA, USA) and 5 μl of viscoelastic solution (10 mg/mL sodium hyaluronate; Healon, Advanced Medical Optics Inc., Santa Ana, CA, USA). Suspension was injected rapidly through a puncture in lower limbus. This method made it possible to achieve long-lasting IOP elevation with chronic damage to RGCs, which can be considered a good simulation of glaucoma. IOP was measured using a tonometer (TonoLab, Icare, Finland) before bead injection at 1, 3 and 7 days after injection and once per week until animals were sacrificed. The obtained values of IOP were comparable with those described in previous study thereby ensuring the reproducibility of the model^23,24^. The right eye was used as a healthy control.

### Preparation of peripheral nerve extract

After anaesthesia (n = 15 rats – 30 nerves) both right and left sciatic nerves were cut at the level of the hip joint. Depending on the predegeneration time – short-term predegeneration (up to 7 days, n = 10 nerves) or long-term predegeneration (up to 14 days, n = 10 nerves), rats were sacrificed by overdose of anaesthetics and decapitation, and the distal stumps of transected sciatic nerves were collected in cold Ringer solution for mammals. Whole intact sciatic nerves (non-predegenerated, n = 10 nerves) were used as a control and subjected to the same procedure. Subsequently, nerves were homogenized (Ultrasonic Processor GE 50, Aldrich, USA) in a buffer (pH 6.4) consisting of 0.1 M morpholinoethane sulfonic acid (MES), 1 mM ethyleneglycol bis (2-aminoethylether)-N,N,N,N′,N′-tetraacetic acid (EGTA), and 0.1 mM methylenediaminetetraacetic acid (EDTA) (all Serva, Heidelberg, Germany), using 2 ml/1 g of nerve tissue. Homogenates were filtered through cotton gauze and filtrates were centrifuged at 700 × g for 10 min. Supernatants were centrifuged at 20 000 × g for 10 min and then ultracentrifuged at 105 000 × g for 120 min to obtain postmicrosomal fractions. All preparation procedures were carried out at 0 °C with addition of 1 mM phenylmethylsulfonyl fluoride (PMSF) (Serva) as a protease inhibitor. Total protein concentration in the final extract was determined by the Bradford method (short-term predegenerated extract 9.67 µg/µl; long-term predegenerated extract 11.45 µg/µl; control extract 6.65 µg/µl). Protein extracts from the nerves predegenerated for 7 days were divided into two portions. One group was used for intravitreal injection in the following experimental groups, according to the day of injection (i.e., 2D – injection on the 2^nd^ day after glaucoma induction, 7D – injection on the 7^th^ day after glaucoma induction, etc.) and follow-up time (i.e., 14 – euthanasia 14 days after glaucoma induction, 21 – euthanasia 21 days after glaucoma induction, etc.): PNE 2D/14_,_ PNE 7D/21, PNE 7D/28, PNE 7D/35, PNE 14D/28, PNE 14D/35 (detailed description in Fig. 7). The remaining amount of short-term predegenerated nerve extracts as well as extracts from the non-predegenerated and long-term predegenerated nerves were analysed by mass spectrometry.

### Intravitreal injection of the peripheral nerve extract

The extract injection (the left eye) was carried out on the 2^nd^ (in PNE 2D/14 group), 7^th^ (in PNE 7D/21, PNE 7D/28, PNE 7D/35 groups) or 14^th^ (in PNE 14D/28, PNE 14D/35 groups) day after induction of experimental glaucoma. Injections of PNE (3 µl/eye) were administered using a 10 µl Hamilton syringe combined with a 6-mm-long 34 G needle. The needle was inserted into the lower quadrant of the eye, approximately 1 mm posterior to the corneal limbus at a depth of approximately 3 mm (i.e., approximately 2/3 of the needle length). Lens scarring was avoided; the extract was injected slowly and the needle was kept in the eye for approximately 2 minutes after injection. In the same way, PBS was injected into the left eye (3 µl/eye) of the control animals to check whether the injection of such a volume of fluid can affect the condition of RGCs. PBS injection time-point (second, seventh or fourteenth day) had no effect on RGC survival; therefore, groups with PBS were divided by the sacrification day only (these subgroups were marked as G/14, G/21, G/28 and G/35).

### Retinal ganglion cells labelling and counting

RGC were labelled with FluoroGold (FG, Biotium, 10 mg) (3% dilution in 10% DMSO-saline) applied stereotactically to both superior colliculi. Five days after the FG injection, rats were deeply anesthetized and transcardially perfused with 0.9% NaCl for 2 min, followed by perfusion with 4% paraformaldehyde (PFA)/0.1 M PBS, pH 7.4, for 10 min. Eyeballs were enucleated and post-fixed in 4% PFA for 3 hours. The whole eyeballs were embedded in paraffin according to a standard protocol, sectioned at a thickness of 5 µm using a microtome (Leica, Germany), collected on slides, deparaffinized and rehydrated. Each eye was divided into two parts. One half of the eye was used for RGC counting, and the other for immunostaining. The number of FG-positive RGC was quantified from 15 serial cross-sections. The cells were counted in each 10^th^ slice, whereby we avoided double counting. The sum of the total number of FG-positive cells from 15 corresponding (from treated and healthy right eye) sections represented different parts of the eye. Counting was performed using a fluorescent microscope (Labophot-2, Nikon) under 20x magnification.

### Immunohistochemistry

Specimens were pre-treated for 5 minutes in 0.01 M citrate buffer, pH = 6.0 at 90 °C, autofluorescence was quenched by 15 min incubation in 0.1 M Glycine-tris-buffered saline (TBS) at room temperature (RT), then unspecific binding was blocked with 10% Normal Goat Serum/0.5% Triton-TBS solution for 30 min at RT. Primary antibodies TBS-dilutions were applied overnight at +4 °C. As primary antibodies, we used rabbit anti-BDNF (Santa Cruz, sc-546, dilution 1:100) and mouse anti-GFAP (Sigma, G3893, dilution 1:500). Species-matching secondary antibodies (AlexaFluor 488 or 594, Thermo, dilution 1:500) were applied for 3 hours at RT. Nuclei were counterstained with 2-(4-amidinophenyl)-6-indolecarbamidine dihydrochloride (DAPI, Sigma). Samples were evaluated using the Zeiss Axio Scope.A1 fluorescent microscope (Zeiss, Oberkochen, Germany).

### Peripheral nerve extract proteomics by mass spectrometry

Bradford assay was performed to determine the protein concentration of cell homogenates. Each of sample consisted of 10 pulled nerves. The same amount of protein (300 μg) was diluted with 50 mM NH_4_HCO_3_ (Sigma Aldrich) to equal concentration (800 μl in total). Diluted samples were reduced with 5 mM Dithiothreitol (Sigma Aldrich) for 30 min at 37 °C with gentle agitation and alkylated with 10 mM iodoacetaminde in dark for 30 min. The samples were digested with sequencing grade trypsin (Promega, Madison, WI, 1:100 enzyme-to-protein ratio) at 37 °C overnight. Samples were quenched with 10% trifluoroacetic acid (TFA) and purified using C-18 microspin columns (The Nest Group Inc., USA). The vacuum-dried samples were finally dissolved in 30 µl 0.1% trifluoroacetic acid and 1% acetonitrile. LC-MS/MS analysis for all samples was performed on LTQ Qrbitrap Elite hybrid mass spectrometer using Xcalibur version 2.0.7 SP1 (Thermo Scientific). Peptide samples were separated by EASY nLC II- reverse phase HPLC nanoflow system (Thermo Scientific) with one C18-A1 trap column (EASY-Column™ 2 cm × 100 μm, 5 μm, 120 Å, Thermo Scientific) and followed an analytical column (EASY-Column™ 10 cm × 75 μm, 3 μm, 120 Å, Thermo Scientific). For each sample 4 µl each were loaded onto the pre-column via autosampler. Peptides were eluted in the analytical column with a gradient of 60 min of buffer B (buffer A: 0.1% formic acid in 98% water and 2% acetonitrile; buffer B: 0.1% formic acid in 98% acetonitrile and 2% water) ranging from 5% to 35%, followed by a 5-min gradient from 35% to 80% of buffer B and 1 min gradient from 80% to 100% of buffer B at a flow rate of 300 nl/min. The analyses were performed in a data-dependent acquisition mode. Data analysis was performed using Proteome Discoverer version 1.4 (Thermo Scientific) together with SEQUEST search engine. The search parameters included precursor mass tolerance of 15 ppm, fragment mass tolerance of 0.8 Da, static peptide modification of carbamidomethyl (+57.021 Da) on Cys, dynamic peptide modification of oxidation (+15.995 Da) on Met residues. Up to 1 missed cleavage was allowed on fully tryptic peptides. For peptide identification, a cut-off with a maximum false discovery rate of 0.05 was used.

### Gene Ontology Analysis

GOrilla was used for gene ontology (GO) enrichment analysis^75,76^. A control sample list (uninjured sciatic nerve) was used as a background list and short-term predegenerated nerve or long-term predegenerated nerve lists were set as a target list to search enriched GO terms for biological process, molecular function and cellular components, with p value cut-offs equal 0.001. The results were sent to REVIGO for subsequent visualization^77^. TreeMap from REVIGO for GOslim biological process and molecular function was created. Each rectangle in TreeMap represents a single cluster. The joined ‘superclusters’ included loosely related terms. The size of the rectangles reflects the p value.

### *Ex vivo* retinal explants culture

Retinal explants culture model was used for neuroprotection screening of MT2 (AH diagnostics, Helsinki, Finland). After animals (n = 8, 4-week old Wistar rats) were sacrificed, eyeballs were enucleated and placed in ice cold solution of 1% penicillin-streptomycin (Gibco). Using a stereomicroscope, anterior segments of eyeballs were removed, and each retina was isolated and divided into two parts. Each half was transferred and placed on a 12-well fitted insert membrane (0.4 µm Millicell tissue culture insert, Millipore, Billerica, MA, USA) with GCL directed on the top. Explants were divided in 3 groups. Ten explants were cultured in standard medium Neurobasal A (Gibco, Carlsbad, CA, USA) containing 2% B-27 supplement (Gibco, Carlsbad, CA, USA), 1% N2 supplement (Invitrogen, Carlsbad, CA, USA), 1% penicillin solution (Santa Cruz) and 0.4% GlutaMax (Gibco, Carlsbad, CA, USA). Another 10 explants were cultured in standard medium additionally supplemented with 1 µg/ml MT2. The last group of 10 explants was cultured in standard medium supplemented with 1 µg/ml MT2 and 1 µg/ml Gentamycin (Gibco). Medium was exchanged every second day, and an additional 2 μl drop of culture medium was placed on explant surface. After 7 days of culture, explants were fixed in 4% PFA and processed for immunostaining and stereology. First, explants were blocked with 20% Normal Goat Serum/0.5% Triton-TBS solution for 30 min at RT. Primary antibody in TBS-dilution was applied overnight at +4 °C. As primary antibody, we used rabbit anti-β3tubulin (Abcam, ab18207, dilution 1:300). Species-matching secondary antibody (AlexaFluor 594, Thermo, dilution 1:500) was applied for 3 hours at RT. Nuclei were counterstained with DAPI (Sigma). Samples were evaluated using fluorescent microscope Zeiss Axio Scope.A1 (Zeiss, Oberkochen, Germany).

### Stereology

We used semiquantitative Stereo Investigator (MicroBrightField Inc, VT, USA) to evaluate density of cells in GCL of retinal explants. To optimise the counting method, corresponding quadrants of retina were selected. For each explant, at least 10 equally distributed frames of dimension 50 × 50 μm were selected automatically and calculated to ensure representation of peripheral and central retina. The density of β3tubulin positive cells in GCL was optimized to explants surface.

### Statistics

Statistical analysis was performed using SPSS 21 (IBM, Armonk, NY, USA). Descriptive statistical results were reported as the mean ± standard deviation. A Kolmogorov–Smirnov test was used to check whether the data were normally distributed. Comparisons between groups were performed using a U-Mann Whitney or independent samples student t-test. For estimated RGC survival, a linear regression model was built. P values < 0.05 were considered statistically significant.

## Electronic supplementary material

Dataset 1

Dataset 2

Supplementary table legends
