# Supplementary material for: Candidate proteins from predegenerated nerve exert time-specific protection of retinal ganglion cells in glaucoma
Source: Sci Rep. 2017 Nov 6;7:14540. doi: 10.1038/s41598-017-14860-5 (PMC5673995; doi:10.1038/s41598-017-14860-5)
Supplement: Supplementary file 2 — Dataset 2 [file 41598_2017_14860_MOESM2_ESM.doc]

| Differences in proteomic features between short-term predegenerated nerves and control ones | | | | |
| --- | --- | --- | --- | --- |
| Function | | | | |
| **GO term** | **Description** | **P-value** | **FDR q-value** | **Enrichment (N, B, n, b)** |
| GO:0044822 | poly(A) RNA binding | 2.46E-5 | 4.66E-2 | 1.15 (1337,180,1037,160) |
| GO:0003723 | RNA binding | 2.77E-5 | 2.63E-2 | 1.13 (1337,219,1037,192) |
| GO:0051015 | actin filament binding | 3.32E-4 | 2.1E-1 | 1.26 (1337,41,1037,40) |
| GO:0005488 | binding | 4.13E-4 | 1.96E-1 | 1.02 (1337,1126,1037,893) |
| GO:0008144 | drug binding | 7.48E-4 | 2.84E-1 | 1.29 (1337,28,1037,28) |
| Process | | | | |
| **GO term** | **Description** | **P-value** | **FDR q-value** | **Enrichment (N, B, n, b)** |
| GO:0006979 | response to oxidative stress | 8.2E-6 | 6.46E-2 | 1.24 (1337,74,1037,71) |
| GO:0032269 | negative regulation of cellular protein metabolic process | 3.98E-5 | 1.57E-1 | 1.17 (1337,129,1037,117) |
| GO:0051248 | negative regulation of protein metabolic process | 7.00E-5 | 1.84E-1 | 1.16 (1337,132,1037,119) |
| GO:0006950 | response to stress | 1.03E-4 | 2.02E-1 | 1.09 (1337,341,1037,289) |
| GO:0045861 | negative regulation of proteolysis | 2.37E-4 | 3.74E-1 | 1.24 (1337,51,1037,49) |
| GO:0034599 | cellular response to oxidative stress | 2.64E-4 | 3.46E-1 | 1.29 (1337,32,1037,32) |
| GO:0048519 | negative regulation of biological process | 9.03E-4 | 1.00E+00 | 1.06 (1337,477,1037,393) |
| GO:0048523 | negative regulation of cellular process | 9.14E-4 | 9.00E-01 | 1.06 (1337,458,1037,378) |

| Differences in proteomic features between long-term predegenerated nerves and control ones | | | | |
| --- | --- | --- | --- | --- |
| Function | | | | |
| **GO term** | **Description** | **P-value** | **FDR q-value** | **Enrichment (N, B, n, b)** |
| GO:0044822 | poly(A) RNA binding | 5.62E-4 | 1.00E+00 | 1.15 (1245,158,889,130) |
| Process | | | | |
| **GO term** | **Description** | **P-value** | **FDR q-value** | **Enrichment (N, B, n, b)** |
| GO:0045861 | negative regulation of proteolysis | 1.32E-4 | 1.00E+00 | 1.31 (1245,47,889,44) |
| GO:0009117 | nucleotide metabolic process | 4.82E-4 | 1.00E+00 | 1.24 (1245,69,889,61) |
| GO:0010466 | negative regulation of peptidase activity | 5.46E-4 | 1.00E+00 | 1.30 (1245,42,889,39) |
| GO:0010951 | negative regulation of endopeptidase activity | 9.50E-4 | 1.00E+00 | 1.30 (1245,40,889,37) |
