# Supplementary material for: Candidate proteins from predegenerated nerve exert time-specific protection of retinal ganglion cells in glaucoma
Source: Sci Rep. 2017 Nov 6;7:14540. doi: 10.1038/s41598-017-14860-5 (PMC5673995; doi:10.1038/s41598-017-14860-5)
Supplement: Supplementary file 3 — Supplementary table legends [file 41598_2017_14860_MOESM3_ESM.pdf]

Candidate proteins from predegenerated nerve exert time-specific protection of retinal ganglion cells in glaucoma

Marita Pietrucha-Dutczak[1*], Adrian Smedowski[1*], Xiaonan Liu[2], Iwona Matuszek[1], Markku Varjosalo[2] and Joanna Lewin-Kowalik[1]

Supplementary table 1. Protein list identified in Mass Spectrometry.

Supplementary table 2. GOrilla gene ontology enrichment analysis for biological process and molecular function with marked p values.
